# Negative Binomial Mixed Models for Analyzing Longitudinal Microbiome Data

**DOI:** 10.3389/fmicb.2018.01683

**Published:** 2018-07-26

**Authors:** Xinyan Zhang, Yu-Fang Pei, Lei Zhang, Boyi Guo, Amanda H. Pendegraft, Wenzhuo Zhuang, Nengjun Yi

**Affiliations:** ^1^Department of Biostatistics, Jiann-Ping Hsu College of Public Health, Georgia Southern University, Statesboro, GA, United States; ^2^Department of Epidemiology and Health Statistics, School of Public Health, Medical College of Soochow University, Suzhou, China; ^3^Department of Biostatistics, School of Public Health, University of Alabama at Birmingham, Birmingham, AL, United States; ^4^Department of Cell Biology, School of Biology & Basic Medical Science, Soochow University, Suzhou, China

**Keywords:** count data, longitudinal study, microbiome, metagenomics, negative binomial mixed model

## Abstract

The metagenomics sequencing data provide valuable resources for investigating the associations between the microbiome and host environmental/clinical factors and the dynamic changes of microbial abundance over time. The distinct properties of microbiome measurements include varied total sequence reads across samples, over-dispersion and zero-inflation. Additionally, microbiome studies usually collect samples longitudinally, which introduces time-dependent and correlation structures among the samples and thus further complicates the analysis and interpretation of microbiome count data. In this article, we propose negative binomial mixed models (NBMMs) for longitudinal microbiome studies. The proposed NBMMs can efficiently handle over-dispersion and varying total reads, and can account for the dynamic trend and correlation among longitudinal samples. We develop an efficient and stable algorithm to fit the NBMMs. We evaluate and demonstrate the NBMMs method via extensive simulation studies and application to a longitudinal microbiome data. The results show that the proposed method has desirable properties and outperform the previously used methods in terms of flexible framework for modeling correlation structures and detecting dynamic effects. We have developed an R package NBZIMM to implement the proposed method, which is freely available from the public GitHub repository http://github.com//nyiuab//NBZIMM and provides a useful tool for analyzing longitudinal microbiome data.

## Introduction

The human microbiome plays an important role in human health and disease. The complex microbiome is inherently dynamic and interacts with the host and the environmental factors over time (Gerber, 2014a). These complex dynamics start from the birth with increasingly richness in the communities of microbiota over time (Palmer et al., 2007; Koenig et al., 2011; Wu et al., 2011; De Muinck et al., 2013; Gerber, 2014a). Recent studies have found that the human microbiome in healthy adults can be altered by various host factors including genotype (Spor et al., 2011; Blekhman et al., 2015; Goodrich et al., 2016a,b), lifestyle such as dietary habit (De Filippo et al., 2010; Wu et al., 2011), physiological status such as aging (Biagi et al., 2010), pathophysiological status (Turnbaugh et al., 2009), and host environment (Dominguez-Bello et al., 2010). The dynamic shifts in compositional features of the microbiome can occur with human diseases such as obesity (Turnbaugh et al., 2006), diabetes (Samuel and Gordon, 2006), infections or inflammatory bowel disease (Frank et al., 2007), and cancers (Holmes et al., 2011). To decipher the relationship between the dynamic changes in microbiome and human diseases, high-throughput sequencing technologies, such as the 16S ribosome RNA (rRNA) gene sequencing or shotgun metagenomics sequencing, have been widely applied in longitudinal microbiome studies (Matsen et al., 2010; Ghodsi et al., 2011; Gilbert et al., 2011; La Rosa et al., 2014).

The metagenomics sequencing data provide valuable resources for investigating the dynamic changes of microbial abundance over time and the associations between the microbiome and host environmental/clinical factors. Multiple recent microbiome studies have employed the longitudinal study designs to address the crucial research question (La Rosa et al., 2014; DiGiulio et al., 2015; Zhou et al., 2015; Ward et al., 2016). Among them, La Rosa et al. (2014) utilized longitudinal analysis of repeated measures data to demonstrate that the dynamic shifts in dominating microbiota of the infant gut from *Bacilli* at birth, giving way to *Gammaproteobacteria*, then *Clostridia* at the end of the first month of life. In another recent published study, Ward et al. (2016) used longitudinal study to address the associations between the dynamic change of the early intestinal microbiome in preterm infants and the occurrence of Necrotizing enterocolitis (NEC) or NEC-associated deaths.

Despite our ability to generate large-scale metagenomics sequencing longitudinal data, many challenges exist in the development of robust and powerful statistical methods and computational tools for properly analyzing and interpreting longitudinal microbiome data. The metagenomics sequencing data has some properties that require tailored analytic tools; these include varied total sequence reads across samples, over-dispersion and zero-inflation. One common way to account for varying total reads is normalization, i.e., conversion of the sequence counts to the relative abundance (or proportion) using the total sum, mean, or median of representative OTUs across all samples (Anders and Huber, 2010; Robinson and Oshlack, 2010; Knights et al., 2011; Wagner et al., 2011; Kostic et al., 2012; Paulson et al., 2013). Several zero-inflated models were proposed to correct for excess zero counts in microbiome measurements, including zero-inflated Gaussian, lognormal, negative binomial, and beta models (Paulson et al., 2013; Peng et al., 2015; Sohn et al., 2015; Xu et al., 2015). On the other hand, the negative binomial regression, which is a standard statistical method for analyzing over-dispersed count observations, has been recently applied to microbiome data (White et al., 2009; Pookhao et al., 2015).

It is even more challenging to analyze longitudinal microbiome count data. In addition to the special features of microbiome data, longitudinal studies possesses two fundamental time-dependent features: (a) time imposes an inherent and irreversible ordering on samples, and (b) samples exhibit statistical dependencies that are a function of time (Gerber, 2014b). Ignoring these properties of longitudinal data and applying statistical tools designed for analyzing static data can result in erroneous conclusions (Gerber, 2014a). Most of the previous studies resort to linear mixed models (LMMs) to account for time-dependent correlations in longitudinal microbiome study designs by treating transformed data as normally distributed responses (Benson et al., 2010; Srinivas et al., 2013; La Rosa et al., 2014; Leamy et al., 2014; Wang et al., 2015). However, using LMMs directly without addressing properties of microbiome data may result in lower power or potential inaccurate results to detect the dynamic effects of microbiota. Chen and Li (2016) developed zero-inflated beta mixed models for analyzing transformed proportions in microbiome longitudinal studies, but did not address time trends and within-subject correlations. Thus, statistical models to account for time series as well as properties of microbiome count data are required for analyzing microbiome data (Spor et al., 2011; Faust et al., 2015; Chen and Li, 2016).

Zhang et al. (2017) have recently developed negative binomial mixed models (NBMMs) for analyzing clustered microbiome data, but have not addressed longitudinal studies yet. We here extend negative binomial mixed models (NBMMs) proposed by Zhang et al. (2017) to analyze longitudinal microbiome count data. The extended NBMMs can include various types of fixed effects and random effects, and can incorporate various correlation structures among observations within the same subjects, thus fully addressing the special properties of longitudinal microbiome count data. We develop an efficient and stable IWLS (iterative weighted least squares) algorithm to fit the extended NBMMs by taking advantage of the standard procedure for fitting linear mixed models. Through extensive simulations, we show that the NBMMs outperform the previously used LMMs in terms of detecting dynamic effects in longitudinal microbiome count data. We also apply our method to a previously published microbiome data to detect significantly dynamic effects of associated taxa. We have implemented the proposed method in the R package NBZIMM, which is freely available from the public GitHub repository http://github.com//nyiuab//NBZIMM and provides a useful tool for longitudinal microbiome studies.

## Methods

### Negative binomial mixed models (NBMMS) for longitudinal microbiome studies

Longitudinal studies collect multiple subjects and measure each subject at multiple time points (i.e., samples). Assume that there are *n* subjects, and subject *i* is measured at *n*_*i*_ time points *t*_*ij*_; *j* = 1, ···, *n*_*i*_; *i* = 1, ···, *n*. For each sample, microbiome data generated by the 16S rRNA gene sequencing or the shotgun metagenomics sequencing consist of counts for numerous taxa at certain taxonomic levels (OTU, species, genus, classes, etc.), *c*_*ijh*_, *h* = 1, ···, *m*, and total sequence read *T*_*ij*_ (also referred to as depths of coverage or library size). We also measure some host clinical/environmental variables for each subject, *X*_*i*_. Table 1 summarizes the data structure for a longitudinal microbiome study. The goal of longitudinal microbiome studies is to detect associations between the microbiome counts and the host variables, and characterize the time trends of microbiome abundance within subjects and between subjects.

**Table 1:** Longitudinal microbiome data structure.

**Subject ID**	**Taxon 1**	**Taxon 2**	**···**	**Taxon *m***	**Total reads**	**Host factors**	**Time variables**
Subject 1	*c_111_*	*c_112_*	*···*	*c_11*m*_*	*T_11_*	*X_1_*	*t_11_*
Subject 1	*c_121_*	*c_122_*	*···*	*c_12*m*_*	*T_12_*	*X_1_*	*t_12_*
Subject 1	*c_131_*	*c_132_*	*···*	*c_13*m*_*	*T_13_*	*X_1_*	*t_13_*
Subject 2	*c_211_*	*c_212_*	*···*	*c_21*m*_*	*T_21_*	*X_2_*	*t_21_*
	· · ·	· · ·	· · ·	· · ·	· · ·	· · ·	· · ·
Subject n	*c_*n*11_*	*c_*n*12_*	*···*	*c_*n*1*m*_*	*T_*n*1_*	*X_*n*_*	*t_*n*1_*

We separately analyze each microbiome taxon, as most existing methods. For notational simplification, we denote *y*_*ij*_ = *c*_*ijh*_ for any given taxon *h*. Since the microbiome count outcome is over-dispersed, we use negative binomial models. We extend negative binomial mixed models (NBMMs) proposed by Zhang et al. (2017) to analyze longitudinal microbiome data by including the time variable and its interaction with the host factor of interest in the model. In the next section, we will further extend NBMMs to account for within-subject correlation structures.

In our NBMMs, the counts *y*_*ij*_ are assumed to follow the negative binomial distribution:

(1) $$\begin{array}{l} y_{ij}\sim \;NB\left(y_{ij}|\mu_{ij},\theta \right)=\frac{\Gamma \left(y_{ij}+\theta \right)}{\Gamma \left(\theta \right)y_{ij}!}\cdot {\left(\frac{\theta }{\mu_{ij}+\theta }\right)}^{\theta }\cdot {\left(\frac{\mu_{ij}}{\mu_{ij}+\theta }\right)}^{y_{ij}} \end{array}$$

where θ is the dispersion parameter that controls the amount of over-dispersion, and μ_*ij*_ are the means. The means μ_*ij*_ are related to the host variables via the logarithm link function:

(2) $$\begin{array}{l} \log\left(\mu_{ij}\right)=\log\left(T_{ij}\right)+X_{ij}\beta +Z_{ij}b_{i} \end{array}$$

where log(*T*_*ij*_) is the offset that corrects for the variation of the total sequence reads, $X_{ij}=\left(1,X_{i},t_{ij},X_{i}^{s}t_{ij}\right)$, $X_{i}^{s}$ is the variable of interest in *X*_*i*_, for example, an indicator variable for the case group and the control group, and*Z*_*ij*_ = (1, *t*_*ij*_); β = (β_0_, β_1_, β_2_, β_3_)^T^ is the vector of fixed effects (i.e., population-level effects), including an intercept β_0_, the effects β_1_ of the host variables *X*_*i*_, the overall time effect β_2_, and the interaction β_3_ between $X_{i}^{s}$ and*t*_*ij*_; *b*_*i*_ = (*b*_0i_, *b*_1i_)^T^ is the vector of random effects (i.e., subject-level effects), including the random intercept *b*_0i_ and the random time effect *b*_1i_. For simplicity, the above model only considers the linear function of *t*_*ij*_. If sample size is large enough, however, we can extend the model to use polynomial functions, for example, (*t*_*ij*_, *t*_*ij*_^2^), or B-spline functions, allowing us to detect arbitrary temporal trends.

The random effects are used to model multiple sources of variations and subject-specific effects, and thus avoid biased inference on the fixed effects. The vector of the random effects is usually assumed to follow a multivariate normal distribution (Pinheiro and Bates, 2000; McCulloch and Searle, 2001):

(3) $$\begin{array}{l} b_{i}\sim \;N\left(0,\text{Ψ}\right) \end{array}$$

where Ψ is the variance-covariance matrix. Ψ can be a general positive-definite matrix that accounts for the correlation of the random covariates. In some applications, however, we can restrict Ψ to special forms of variance-covariance matrices that are parameterized by fewer parameters. For example, we may assume that the random effects are independent, in which case Ψ is a diagonal matrix.

### Accounting for within-subject correlations and IWLS algorithm for fitting the NBMMS

The IWLS (Iterative Weighted Least Squares) algorithm developed by Zhang et al. (2017) can be used to fit the above NBMMs. The basic idea of the IWLS algorithm is to iteratively approximate the negative binomial mixed model by a linear mixed model. However, Zhang et al. (2017) restricts the within-subject errors in the linear mixed model to be independent, and thus ignores special within-subject correlation structures. For longitudinal data, however, samples within the same subject are usually correlated. Thus, we extend the model by relaxing the assumption of independent within-subject errors to account for special within-subject correlation structures:

(4) $$\begin{array}{r} z_{ij}=\log\left(T_{ij}\right)+X_{ij}\beta +Z_{ij}b_{i}+w_{ij}^{-1/2}e_{ij},b_{i}\sim \;N\left(0,\text{Ψ}\right),\\ e_{i}={\left(e_{i1},\cdots \;,e_{in_{i}}\right)}^{\prime }\sim \;N\left(0,\sigma^{2}R_{i}\right) \end{array}$$

where *z*_*ij*_ and *w*_*ij*_ are the pseudo-responses and the pseudo-weights, respectively, that depend on $\log\left(T_{ij}\right)+X_{ij}\hat{\beta }+Z_{ij}{\hat{b}}_{i}$ and $\hat{\theta }$ as described in Zhang et al. (2017), and *R*_*i*_ is a correlation matrix, which describes dependence among observations, Pinheiro and Bates (2000) describes several ways to specify the correlation matrix *R*_*i*_, all of which can be incorporated into our NBMMs. For longitudinal studies, a common choice of *R*_*i*_ is autoregressive of order 1, AR(1), or continuous-time AR(1).

We extend the IWLS algorithm developed by Zhang et al. (2017) to fit the proposed NBMMS with correlation structures. The algorithm alternatively updates the dispersion θ and the parameters in the linear mixed model (4). Given the estimates of β and *b*, we update the dispersion parameter θ by maximizing the negative binomial likelihood using the standard Newton-Raphson algorithm, and then calculate the pseudo-responses and the pseudo-weights. We then fit the linear mixed model (4) using the standard method as implemented in the core package **nmle** in R. At convergence of the algorithm, we get the maximum likelihood estimates of all the fixed effects β_*k*_ and their confidence intervals from the final linear mixed model. We then can test H_0_: β_*k*_ = 0 following the linear mixed model framework.

### R package for implementing the proposed method

We have created the function **glmm.nb** for setting up and fitting the proposed NBMMs, which is part of the R package **NBZIMM**. The function **glmm.nb** works by repeated calls to the function **lme** for fitting linear mixed models in the recommended package **nlme** in R, and allows for any types of random effects and within-subject correlation structures as described in the package **nlme**. The outputs from the function **glmm.nb** can be summarized by functions in **nlme**. The package **NBZIMM** is freely available from the public GitHub repository http://github.com//nyiuab//NBZIMM.

## Results

### Simulation studies

#### Simulation designs

We performed extensive simulations to evaluate the proposed methods. We extended the simulation framework of Zhang et al. (2017) to simulate longitudinal microbiome counts from negative binomial distributions and incorporate time covariates, random effects and within-subject correlation structures.

Our simulation studies employed a case-control longitudinal study design with four different settings. All the four simulation settings followed a two-level longitudinal study, where all individuals (subjects) were from two groups (i.e., case or control) and multiple samples were measured at several time points for each individual. For all the settings, we simulated (*n* =) 50, 100 or 150 individuals, half of which were cases, and included three fixed covariates: a binary case-control indicator variable *x*_*i*_, a continuous time variable *t*_*ij*_, and their interaction. We denote the fixed effects of these three covariates by (β_1_, β_2_, β_3_). The time points, random effects, and within-subject correlation structures were set as follows:

1. Setting A: 5 time points for each individual, only random intercept, and no within-subject correlation;
2. Setting B: 10 time points for each individual, only random intercept, and the within-subject correlation was autoregressive of order 1, AR(1);
3. Setting C: 5 time points for each individual, two random effects (i.e., random intercept and time effect), and no within-subject correlation;
4. Setting D: 4 or 5 different time points for individuals, only random intercept, and no within-subject correlation;

To minimize possible bias and yield reasonable count values that are similar to real microbiome data, we randomly generated the parameters in the model from reasonable ranges at each simulation replication (Zhang et al. 2017), which are described as follows:

1. The values, log(*T*_*ij*_) + β_0_, control the means of simulated counts when all the effects are zero, where β_0_ is the fixed intercept. We set β_0_ = −7 and randomly sampled log(*T*_*ij*_) from the range [7.1, 10.5]. In this case, log(*T*_*ij*_) + β_0_ were in the range [0.1, 3.5], which yield counts similar to real microbiome data;
2. The dispersion parameter θ were uniformly sampled from the range [0.1, 5], which yield highly or moderate over-dispersed counts;
3. To evaluate false positive rates, the fixed effects β_1_, β_2_ and β_3_ were all set to be zero. To evaluate empirical powers, we considered four scenarios: a) β_1_ and β_2_ were set to 0, and β_3_ was sampled from [0.2, 0.35]; b) β_1_ and β_2_ were set to 0, and β_3_ was sampled from [0.35, 0.8]; c) β_1_, β_2_ and β_3_ were all sampled from [0.2, 0.35]; d) β_1_, β_2_ and β_3_ were all sampled from [0.35, 0.8];
4. The random effects *b*_0i_ and *b*_1i_ were generated from N(0, τ^2^), where τ was randomly drawn from the range [0.5, 1];
5. The correlation coefficient ρ for AR(1) correlation was sampled from [0.1, 0.5], and the AR(1) correlation was generated by the function *arima.sim()* from R package *stats*;
6. The standard deviation σ was sampled from [0.1, 0.5];

The ranges of all the parameters used in the simulation are summarized in Table 2.

**Table 2:** Parameter ranges in simulation studies.

**Parameter**	**Range**
log(*T_*ij*_*) + β_0_	Unif(0.1, 3.5)
Dispersion parameter θ	Unif(0.1, 5)
Fixed effects β_1_, β_2_, β_3_ (false positive rate)	0, 0, 0
Fixed effects β_1_, β_2_, β_3_ (power of interaction)	0, 0, Unif(0.2, 0.35) or Unif(0.35, 0.8)
Fixed effects β_1_, β_2_, β_3_ (power of both β_1_ and β_3_)	All from Unif(0.2, 0.35) or Unif(0.35, 0.8)
Standard deviation τ	Unif(0.5, 1)
Correlation ρ	Unif(0.1, 0.5)
Standard deviation σ	Unif(0.1, 0.5)

In all the four simulation settings, the procedure was repeated 10,000 times. At each replication, the parameters were sampled from the ranges described above. There were two hypotheses of interests to be tested, i.e., the group main effect β_1_ = 0 and the group by time interaction β_3_ = 0. Both empirical power and false positive rate for testing the hypotheses were calculated under significance level at 0.05. The empirical power and false positive rate were defined as the proportions of detecting non-zero and zero effects over the simulation replications, respectively. We compared the proposed NBMMs with the linear mixed model with the arcsine square root transformation, $arcsine\left(\sqrt{y_{ij}/T_{ij}}\right)$, as the response, denoted by LMM arcsin.

#### Simulation results

**Figure 1:**
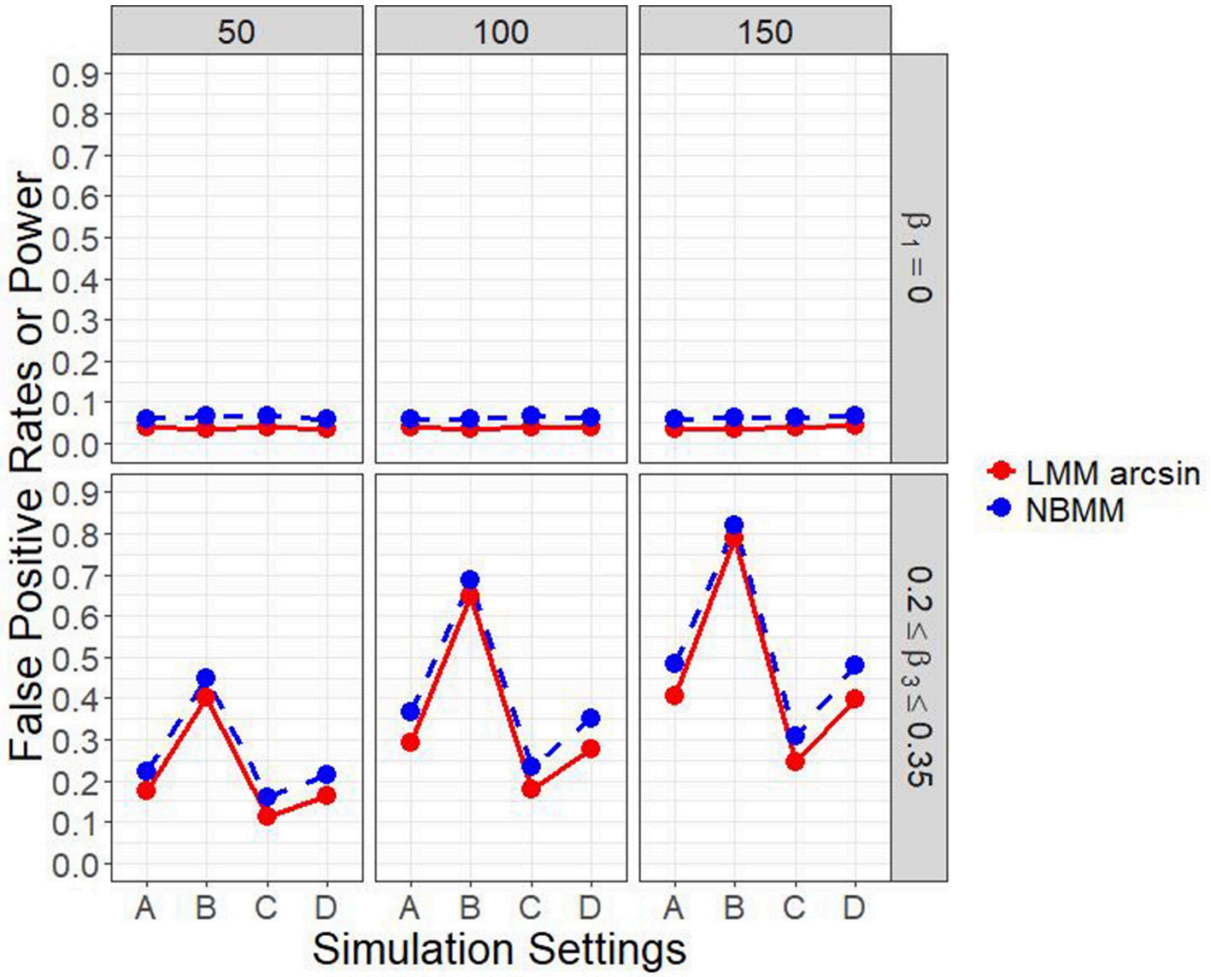
Empirical power of interaction term and false positive rates of main effect in all four simulation settings.

Figure 1 and Figure A.1 show the empirical power to detect the group by time interaction under the four different simulation settings, when the group main effect was set to zero. The power was affected by the sample size. It can be clearly seen that the proposed method performed consistently better than the LMM arcsin method across almost all the scenarios. The second setting was set to represent time-series structure in longitudinal data with 10 measurements for each individual, and thus had the largest power among all the four settings. It was shown that the first setting had higher power than the third setting, on the other hand, a similar performance in power compared with the fourth setting.

**Figure 2:**
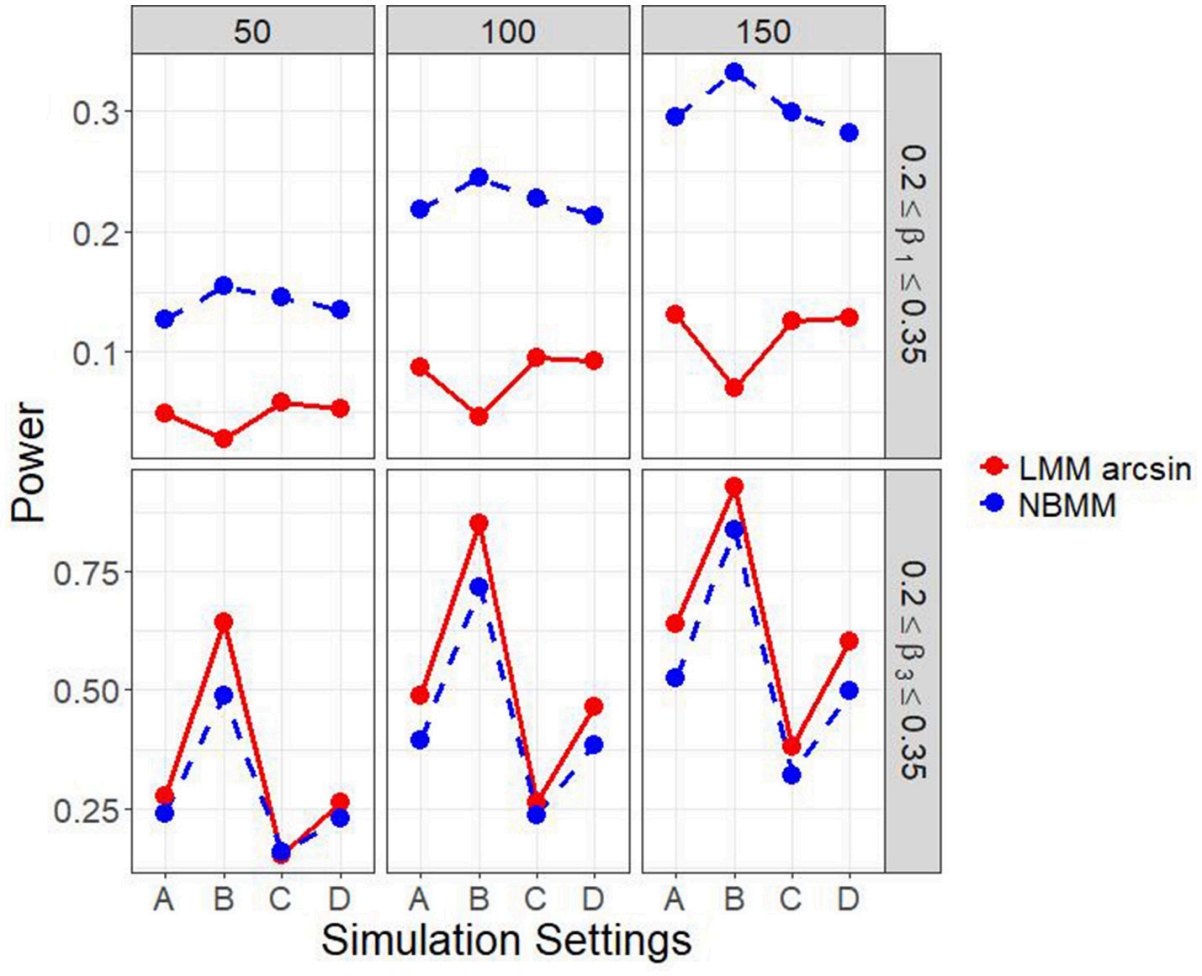
Empirical power of both interaction term and main effect in all four simulation settings.

**Figure 3:**
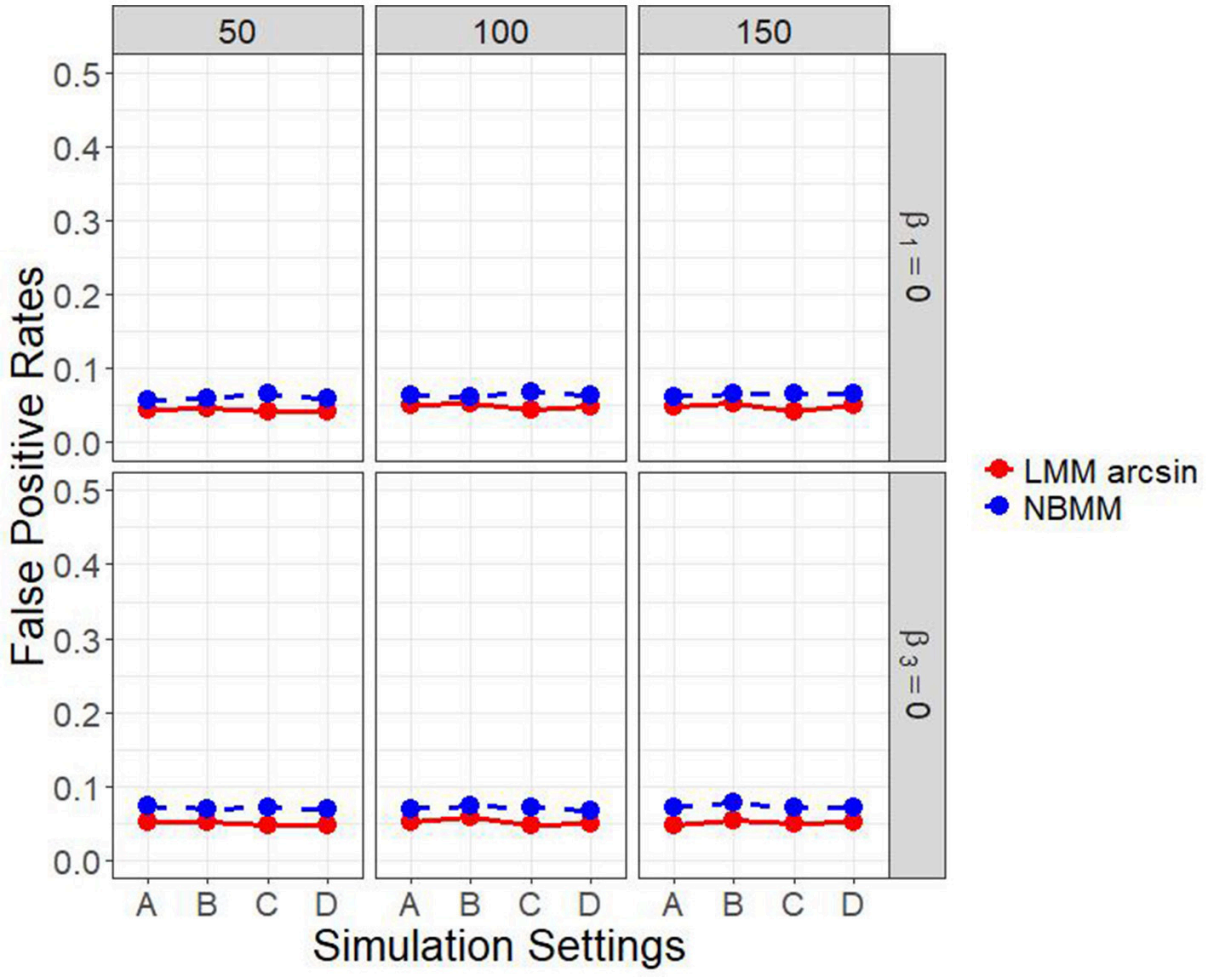
False positive rates of both interaction term and main effect in all four simulation settings.

It is of interest to detect both the group main effect and the group by time interaction. Therefore, in another set of parameter settings, we targeted to detect both the group main effect and the group by time interaction. Figure 2 and Figure A.2 show the empirical power to detect both the group main effect and the group by time interaction under the four different simulation settings. The results showed that the LMM arcsine method resulted in a slightly higher power in detecting interaction term than our proposed method across all the scenarios. However, it showed an extreme low power close to alpha level in detecting the group main effect across all the scenarios. It inferred that LMM arcsine method is not an appropriate approach to be used when the group main effect and the group by time interaction effect are both nonzero. Figure 3 displays the false positive rates for detecting both the group main effect and interaction effect. For all the four simulation settings, the false positive rates were well controlled under all the scenarios.

### Application to temporal and spatial pregnant data

We applied our method to a public microbiome data from a longitudinal study to investigate the bacterial taxonomic composition for pregnant and postpartum women by DiGiulio et al. (2015). This case-control longitudinal study included 49 pregnant women, 15 of whom delivered preterm. The discovery data was consisted with 40 of those women. Among those 40 women, they collected 3,767 specimens prospectively and weekly during gestation and monthly after delivery from the vagina, distal gut, saliva, and tooth/gum. The specimens were analyzed for bacterial taxonomic composition. The final dataset contained a total of 1271 taxa from 3432 specimens which were identified for pregnant women delivered at term and preterm. Detailed information about population and material is available in DiGiulio et al. (2015). Clinical data included race, weeks/days when the samples were obtained, way of delivery, and household income level were acquired. The public processed OTU data available from the study is from species level. The clinical data for the validation dataset for the rest of 9 pregnant women is not available.

We used the proposed NBMMs and the linear mixed models (LMMs) with the arcsine square root transformations to detect associations between delivery term and vaginal bacteria taxa composition during pregnancy. The host factor in the analysis was defined as two groups with patients who delivered at preterm vs. term. The patients who delivered at marginal term were excluded from the analysis. Only specimens collected in vaginal during pregnancy were included in the analysis. Meanwhile, according to the original paper, the samples could be divided to 5 Vaginal Community State Types. Only samples with community state type 4 were analyzed in the original paper. To be consistent, we followed the same criteria for sample filtering. The sample size in the final analysis was 103. We included 58 taxa with zero proportion greater than 0.25 for 103 samples in our analysis. The real data and the R code for our analysis are available from the GitHub page: https://abbyyan3.github.io//NBZIMM-tutorial/NBZIMM_NBMMs_Longitudinal.html.

To compare the abilities of LMMs and NBMMs in detecting the static and dynamic association between host factor and vaginal bacterial taxa composition, we used the following four different models:

1. Model A: the host factor as fixed effect only, no host factor and time interaction term, only random intercept;
2. Model B: the host factor as fixed effect only, no host factor and time interaction term, two random effects (i.e., random intercept and time effect);
3. Model C: the host factor, time, host factor and time interaction term as fixed effects, only random intercept;
4. Model D: the host factor, time, host factor and time interaction term as fixed effects, two random effects (i.e., random intercept and time effect);

We summarized the number of significant taxa and calculated the rate of significant taxa detected by LMMs and NBMM each using Model A-D at alpha level at 0.05 (Table 3). In model A and model B, the numbers of detected significant taxa were substantially less than the numbers from model C and model D. It inferred that failing to incorporate the host factor and time interaction term as fixed effect in the model will largely affect our ability to detect shifts in microbiome studies. Meanwhile, it showed that our NBMMs is capable in detecting more significant taxa than LMMs. Consistent differences have also been found at different significance levels, like 0.01 and 0.001.

**Table 3:** Significant taxa rates detected in four models with LMMs and NBMMs.

		**Alpha Level**	**0.05**
Model 1	Test of β_1_	LMMs	0.034483
		NBMMs	0.068966
Model 2	Test of β_1_	LMMs	0.034483
		NBMMs	0.12069
Model 3	Test of β_1_	LMMs	0.12069
		NBMMs	0.224138
	Test of β_3_	LMMs	0.137931
		NBMMs	0.275862
Model 4	Test of β_1_	LMMs	0.137931
		NBMMs	0.206897
	Test of β_3_	LMMs	0.137931
		NBMMs	0.293103

**Figure 4:**
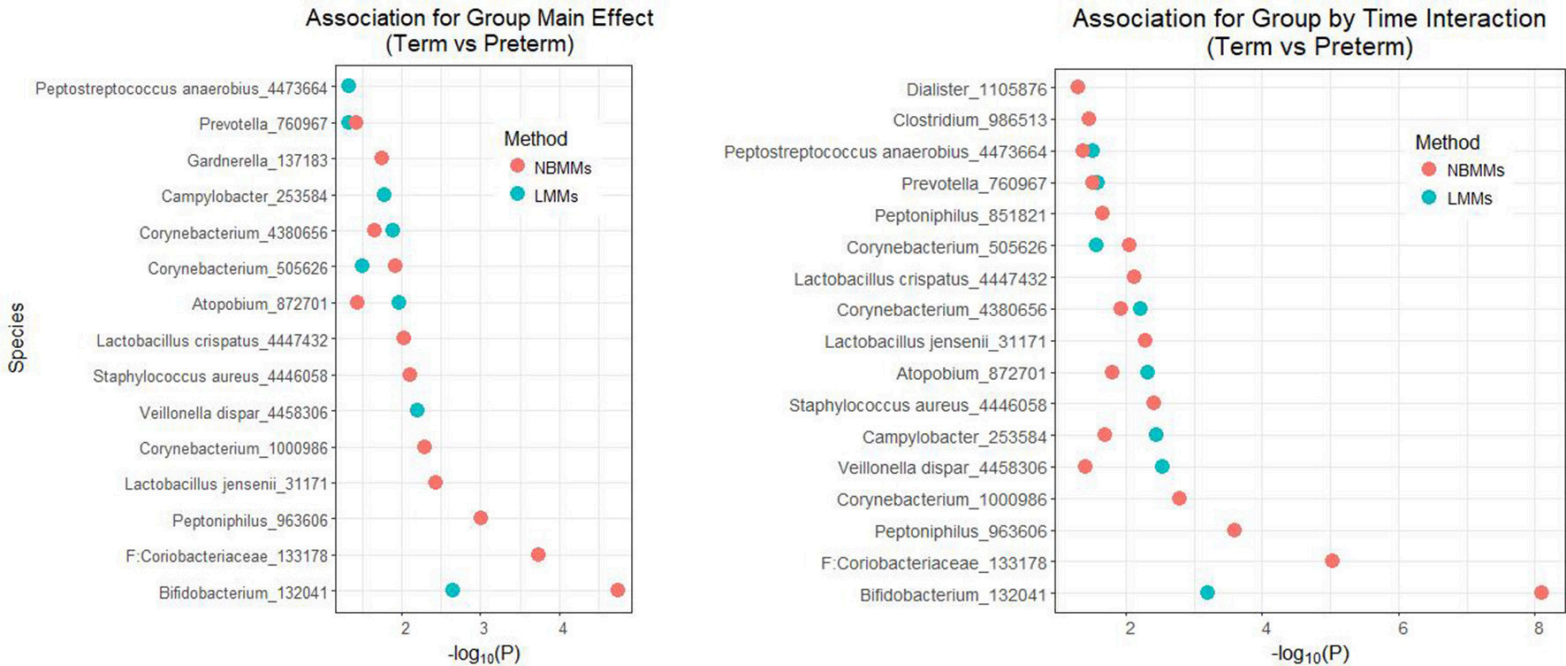
The analyses of NBMMs and LMM: minus log transformed *p*-values for the significant differentially abundant taxa at the 5% significance threshold between term and preterm groups for species level; the left panel shows the minus log transformed *p*-values for association of group main effect, and the right panel shows the minus log transformed *p*-values for association for group by time interaction.

Figure 4 shows the significant features of species level in the model with the host factor and the host factor and time interaction term both at the 5% significance threshold and their minus log transformed *p*-values for NBMMs and LMMs. It showed that NBMMs could discover more species than LMMs in detecting both static association (with host factor term) and dynamic association (with host factor and time interaction term). To compare our analysis results with the published results in DiGiulio et al. (2015), we found that the original paper made two extreme assumptions to the longitudinal study as completely independent or averaged over samples for each subject. The top identified taxa overlapped between our NBMMs with the original paper included *Gardnerella_137183, Lactobacillus jensenii_31171, Staphylococcus aureus_4446058, Lactobacillus crispatus_4447432, Prevotella_760967, Dialister_1105876*. In summary, our NBMMs method is not only a statistical valid method without making extreme assumptions and data transformation, but also detected more significant taxa and yielded much smaller *p*-values than the LMMs, showing that the proposed method could be more powerful than the conventional LMMs.

## Discussion

The main research interest in longitudinal microbiome study is to detect the associations between host clinical/environmental factors and the dynamic shifts in microbiome composition while accounting for sources of heterogeneity and dependence in microbiome measurements. To study the dynamic composition of microbiome, many studies collect samples with temporal structures (Hill et al., 2010; Morrow et al., 2013; Srinivas et al., 2013; La Rosa et al., 2014; Leamy et al., 2014; Faust et al., 2015; Wang et al., 2015; Zhou et al., 2015). These longitudinal studies enable us to study the inherent dynamic properties in microbiome data which have provided extraordinary opportunities to elucidate the true roles of the microbiome in health and disease states and to develop new diagnostics and therapeutic targets (Knights et al., 2011; Segata et al., 2011; Virgin and Todd, 2011; Collison et al., 2012). Accurately identifying and understanding these associations is critical to further predict the probabilities of disease with the identified taxa or biomarkers. However, the traditional methods of using LMMs to model longitudinal data fail to address the count data features in microbiome data. Our simulation studies revealed the impact of the specific features on the microbiome data, showing that ignoring those features can substantially reduce the power for detecting the effects of host clinical/environmental factors with dynamic effects, thus leading to biased and false inferences. We extended our previously proposed negative binomial mixed model (NBMMs) specifically to directly analyze longitudinal microbiome count data without data transformation.

The previously proposed NBMMs (Zhang et al., 2017) have demonstrated its superior ability in family structured clustered microbiome count data. The proposed NBMMs directly model microbiome counts generated by the 16S rRNA gene sequencing or the shotgun sequencing with an efficient IWLS algorithm (Schall, 1991; Breslow and Clayton, 1993; McCulloch and Searle, 2001; Venables and Ripley, 2002). It not only addresses statistical challenges of over-dispersion and varied total reads in microbiome count data, but also accounts for correlation among the observations. Our simulations and real data analysis also show that our algorithm is stable and efficient (Zhang et al., 2017). Meanwhile, the IWLS algorithm is an extension of a commonly used procedure for fitting GLMs and GLMMs which allows us to model non-constant variances or special correlation structures. Therefore, by extending the NBMMs to analyze longitudinal microbiome count data, we illustrated the capability of our proposed NBMMs to handle complex longitudinal study design, such as to include time in the random slope model or to account for the auto-regressive residual correlation in time-series data. Our simulations indicate that our proposed approach is flexible to handle complex structured longitudinal data, allowing for incorporating any types of random effects and within-subject correlation structures (Pinheiro and Bates, 2000; McCulloch and Searle, 2001). In the simulations, our proposed approach outperformed LMMs consistently.

We also applied our method to a previously published data set. The purpose of the real data is to detect host factors that associated with dynamic compositional features of the microbiome (Leamy et al., 2014). Notably, by applying our NBMMs to the temporal and spatial dataset from DiGiulio et al. (2015), the goal of our analysis was to detect taxa that are significantly associated with dynamic change in compositional microbiome between termed and preterm pregnancy. Our proposed method detected the same species *Gardnerella_137183, Lactobacillus jensenii_31171, Staphylococcus aureus_4446058, Lactobacillus crispatus_4447432, Prevotella_760967, Dialister_1105876*, as in the original paper. In the original paper, they made two extreme assumptions to the longitudinal study as completely independent or averaged over samples for each subject. Our NBMMs, on the other hand, does not make any extreme assumption and is more statistically valid. Nevertheless, we still identified overlapped species as in the original paper, showing NBMMs picked out the significant species under extremes as well. Our NBMMs method detected more significant taxa and yielded much smaller *p*-values than the LMMs, showing that the proposed method could be more powerful than the conventional LMMs. Furthermore, comparing the species identified in the real data using LMMs and NBMMs, we found that the species identified by NBMMs only are mostly overlapped with the original paper. It inferred that the transformation of count data could potentially lead to misleading information and interpretation. One potential limitation of our NBMMs is that it is not designed to explicitly handle zero-inflation and we recommend it as future work. Even though, our NBMMs has shown it outperformed LMMs in longitudinal microbiome study in terms of power and accurate interpretation. It is also directly applicable to be used as an analytic tool in longitudinal RNA-seq study.

## Author contributions

NY design the study, develop the method and the software, and participate in writing the paper; XZ simulation study, real analysis, and draft the manuscript; Y-FP design the study, real data analysis, and participate in writing the paper; LZ design the study, and participate in writing the paper; BG design the simulation and real data analysis; AP participate in revising the manuscript; WZ real data analysis, and participate in revising the manuscript.

### Conflict of interest statement

The authors declare that the research was conducted in the absence of any commercial or financial relationships that could be construed as a potential conflict of interest.
