# Supplementary material for: Negative Binomial Mixed Models for Analyzing Longitudinal Microbiome Data
Source: Front Microbiol. 2018 Jul 26;9:1683. doi: 10.3389/fmicb.2018.01683 (PMC6070621; doi:10.3389/fmicb.2018.01683)
Supplement: Supplementary file 1 [file Presentation_1.PDF]

Supplementary Material

Negative Binomial Mixed Models for Analyzing Longitudinal Microbiome Data

Supplementary Figures

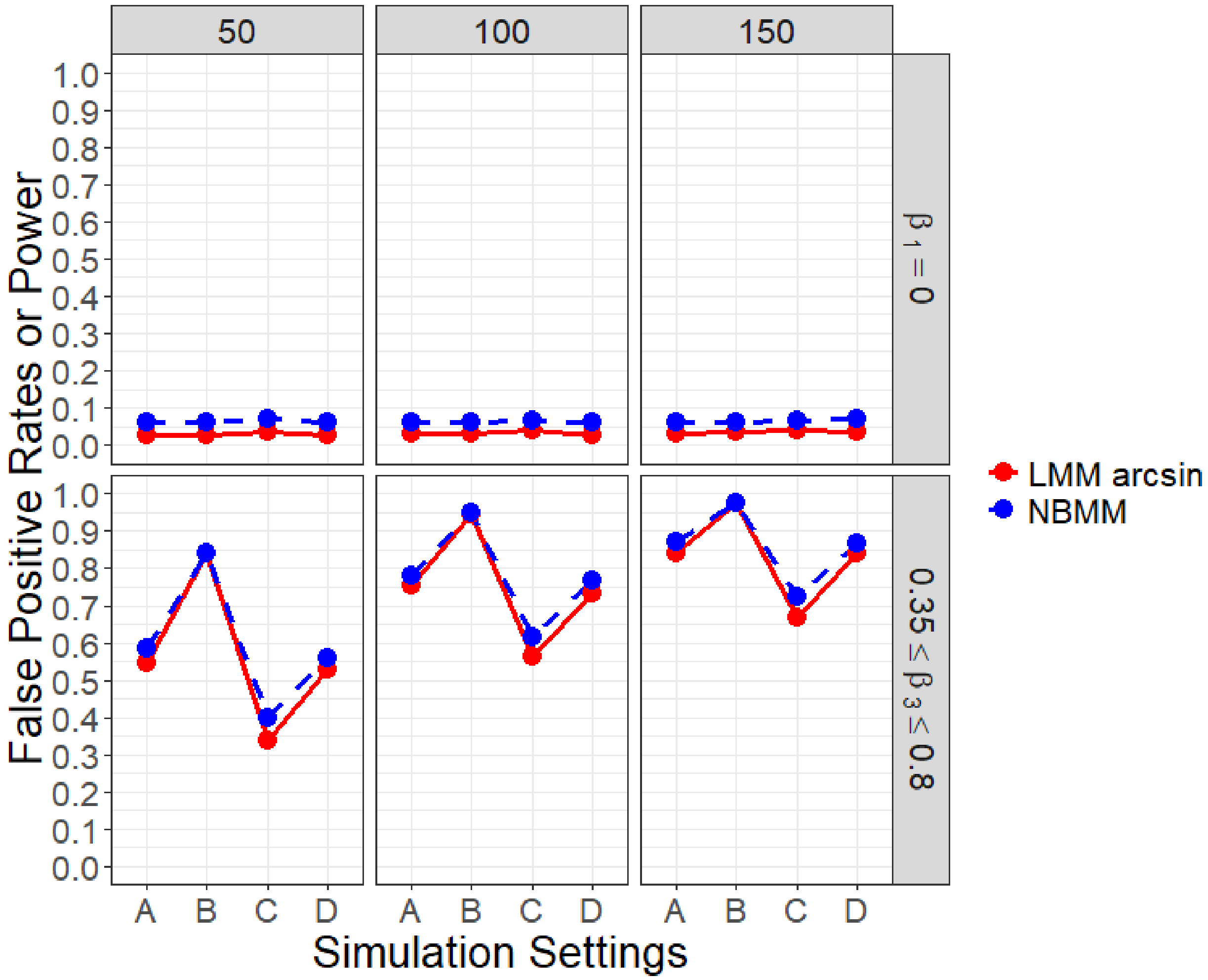

Figure A.1 Empirical power of interaction term and false positive rates of main effect in all Four Simulation Settings.

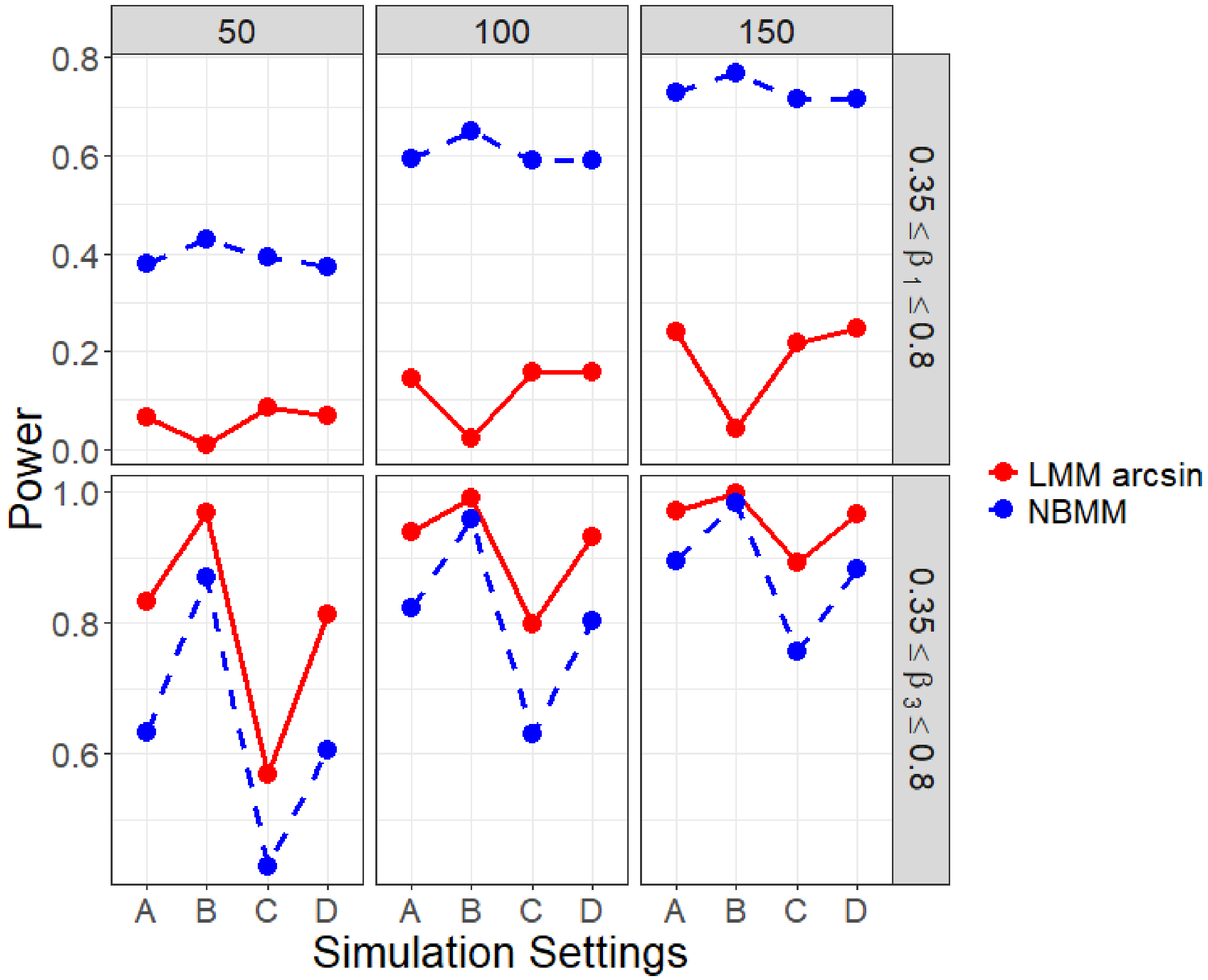

Figure A.2 Empirical power of both interaction term and main effect in all Four Simulation Settings.
